# Mitochondrial genome of *Diploderma micangshanense* and its implications for phylogeny of the genus *Diploderma*

**DOI:** 10.1080/23802359.2021.1882908

**Published:** 2021-03-11

**Authors:** Yanping Li, Yongming Wang, Yinlong Bai, Yunyun Lv, Jianli Xiong

**Affiliations:** a Key Laboratory of Sichuan Province for Fishes Conservation and Utilization in the Upper Reaches of the Yangtze River, College of Life Sciences, Neijiang Normal University, Neijiang, China; b College of Animal Science and Technology, Henan University of Science and Technology, Luoyang, China

**Keywords:** *Diploderma micangshanense*, mitochondrial DNA, phylogenetic analysis

## Abstract

The lizard *Diploderma micangshanense*, which belongs to the family Agamidae is endemic to China. Here, we determined the complete mitogenome of *D. micangshanense* using an Illumina Hiseq X Ten sequencer. This mitogenome’s structure is a typical circular molecule of 16,467 bp in length, consisting of 13 protein-coding genes, 22 transfer RNA genes, 2 ribosomal RNA genes, and a control region. The overall base composition of *D. micangshanensis* is 34.1% A, 23.64% T, 13.62% C, and 28.64% G with a slight AT bias of 57.74%. Most mitochondrial genes except *ND6* and seven tRNAs were encoded on the heavy strand. Notably, the *trnP* gene was encoded on the heavy strand instead of its typical light strand position, providing an example of gene inversion in vertebrate mitogenomes. Phylogenetic analysis indicated that *D. micangshanensis* had a close relationship with *D. zhaoermii*.

## Introduction

Animals in the genus *Japalura sensu lato* are important components of species diversity in Agamidae, and are widely distributed in East Asia and the Himalayas (Manthey 2008). Recently, the current taxonomy of *Japalura sensu lato* has been redefined as four genera, including *Japalura sensu stricto*, *Pseudocalotes*, *Cristidorsa*, and the resurrected genus *Diploderma* (Wang et al. 2019). Almost all the species of the original *Japalura sensu lato*, have been assigned to *Diploderma*, except for *J. bapoensis*, which has been reclassified to genus *Pseudocalotes*, and two species recorded from southern Tibet, *J. andersoniana* and *J. tricarinata*, which are still remain in *Japalura sensu stricto*. Currently, there are 27 species belonging to *Diploderma*; of these, 22 are specifically distributed in China, while *D. polygonatum* is distributed in China and Japan, and the remaining four species are distributed in Vietnam, Myanmar and mainland Southeast Asia.

*Diploderma micangshanensis* is distributed in Sichuan, Shaanxi, Shanxi, Gansu and Henan Provinces. It bears Least Concern status on the International Union for the Conservation of Nature (IUCN) Red List of Threatened Species (IUCN 2020). However, the available genetic data for this species remains scarce. Mitochondrial DNA has many valuable features including relatively conserved gene content and organization, lack of genetic recombination, maternal inheritance, and relatively fast evolutionary rate. Hence, partial or complete mitochondrial genes have been used for species identification (Hebert et al. 2003; Chambers and Hebert 2016), and to determine molecular phylogenetic and evolutionary relationships (Leavitt et al. 2017; Medina et al. 2018; Shahamat et al. 2020). In this study, we assemble and annotate the mitochondrial genome of *D. micangshanensis*, and determine its genomic structure and base composition. We also reconstruct the phylogenetic relationships within the genus *Diploderma* using the mitochondrial sequence *ND2* obtained here and from NCBI. This study not only improves understanding of genomic information and phylogenetic of *Diploderma*, but is also conducive to the conservation genetics of *D. micangshanensis. *

## Materials and methods

### Sample collection

Samples were collected from Luoning County, Henan Province, China (34°16′48″N, 111°43′5″E). Muscle samples were preserved in 95% ethanol, and voucher samples were deposited in the Museum of Henan University of Science and Technology (contact with Jianli Xiong, xiongjl@haust.edu.cn) under the voucher number HNUSTM20200824. Sampling was performed according to Chinese animal protection laws.

### DNA extraction and sequencing

Genomic DNA was extracted from muscle tissue using a DNeasy Blood & Tissue Kit (QIAGEN, Hilden, Germany). DNA integrity, purity and concentration were assessed with an Agilent 5400 fragment analyzer (Agilent Technologies, Santa Clara, CA, U.S.A.). After the DNA sample was qualified, and the template size is 21.578 ng/ul, it was randomly disrupted with a Covaris ultrasonicator (Covaris Inc., Woburn, MA, USA), and then the library was constructed through several steps: end repair and phosphorylation, adding A-tailing, ligating index adapter, purification, denaturing and PCR amplification. After the library was constructed, a Qubit 2.0 (Life Technologies, Singapore) was used to quantify and dilute the library. We then employed an Agilent 2100 Bioanalyzer (Agilent) to detect inserted fragments in the library. Finally, the effective concentration of the library was accurately quantified by q-PCR to ensure the library quality. After that, different libraries were pooled into the flow cell according to the effective concentration and target drop-off data. Illumina paired-end sequencing was conducted with an Illumina Hiseq X Ten sequencer (Illumina, San Diego, CA, USA).

### Mitochondrial genome assembly and annotation

The raw data contained adapter information, low-quality bases, and undetected bases (indicated by N), which would interfere with subsequent analysis. We therefore filtered the raw data using the following criteria: (1) Filtered out reads containing adapter sequences; (2) removed paired reads, when the content of N in a single-ended sequence exceeded 10%; (3) Base with quality no more than 5 was regarded as low-quality base based on phred + 33. If in a sequence more than half were low-quality bases, this sequence, along with the paired one was discarded. The remaining clean data was used for mitochondrial genome assembly with MitoZ v.2.4 using default parameters (Meng et al. 2019). Clade and required taxa were set to Chordata and Japalura, respectively. The assembled genome was annotated using MitoZ v.2.4 with *Diploderma flaviceps* (NC_039541.1) as the reference (Liu et al. 2019).

### Phylogenetic analysis and genetic distance estimate

To examine the evolutionary status of *D. micangshanensis*, we used ND2 regions of *Diploderma* species for phylogenetic inference with *Pseudocalotes flavigula* as the outgroup. Of the 27 valid species currently recognized in genus *Diploderma*, we covered 19 species for which ND2 sequences were available so far, including *D. zhaoermii* (*n* = 1), *D. micangshanensis* (*n* = 3), *D. varcoae* (*n* = 1), *D. dymondi* (*n* = 1), *D. swinhonis* (*n* = 1), *D. polygonatum* (*n* = 1), *D. makii* (*n* = 1), *D. luei* (*n* = 1), *D. brevipes* (*n* = 1), *D. splendidum* (*n* = 1), *D. flaviceps* (*n* = 1), *D. yunnanense* (n = 2), *D. chapaense* (n = 2), *D. yulongense* (*n* = 1), *D. batangensis* (*n* = 2), *D. vela* (*n* = 1), *D. slowinskii* (*n* = 1), *D. laeviventre* (*n* = 1), and *D. swild* (*n* = 1). Multiple codon-based alignments were conducted with MEGA v.7 (Kumar et al. 2016) with the MUSCLE module, and each alignment was further manually corrected. Firstly, the genetic distances of these 19 species were calculated using Kimura 2-parameter (K2P) model (Kimura 1980) with MEGA v.7, which showed the intraspecific genetic distance, and confidence was assessed with 1000 bootstrap replications. Subsequently, we predicted the best nucleotide substitution model using jModeltest v.2 (Darriba et al. 2012) with Bayesian Information Criterion (BIC). We used IQ-tree v.1.6.2 (Nguyen et al. 2015) to construct phylogenetic topologies based on maximum likelihood (ML) and Bayesian inference (BI), using an HKY + F + I + G4 model. Node support of the trees was inferred by bootstrapping with 1000 replications. Trees were graphically visualized and edited with FigTree v1.4.0 (Rambaut and Drummond 2012).

## Results and discussion

**Figure 1.**
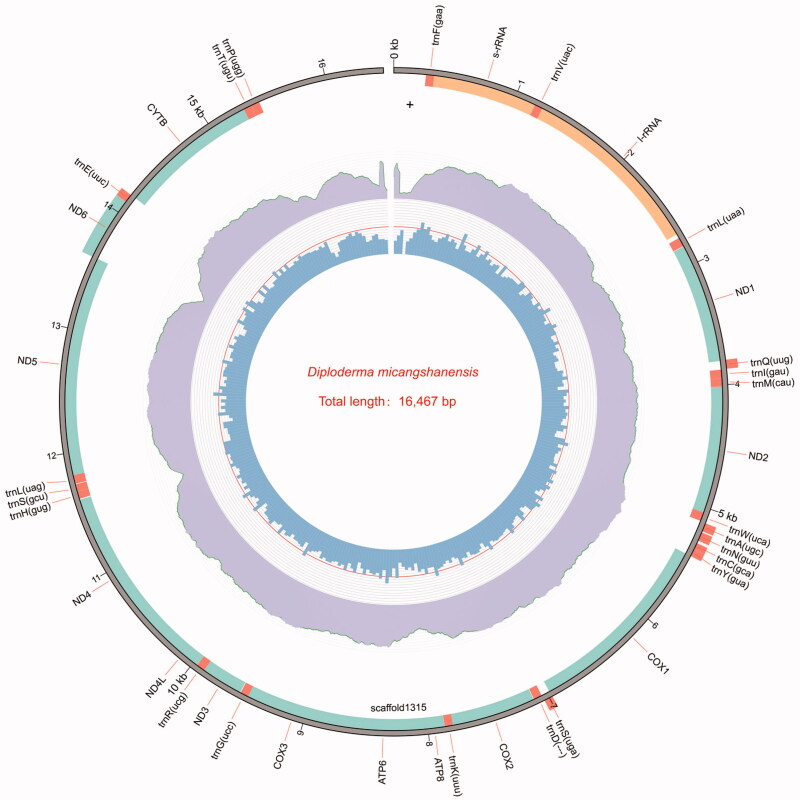
The complete mitochondrial genome of *Diploderma micangshanensis*. The inner blue bars indicate the GC content, the middle circle indicates sequencing depth distribution, and the outermost layer is the gene structure, where orange-yellow indicates the rRNA genes, orange-red indicates the tRNA gene, and light green indicates the 13 protein-coding genes.

A total of 22,652,258 raw reads was generated and it has been deposited to NCBI database (see additional details in Data availability statement). After assembly, the complete mitogenome of *D. micangshanensis* was obtained (accession number: MW242820), with a total length of 16,467 bp, similar to other agamid species (Liu et al. 2019). The mitogenome of *D. micangshanensis* consists of 13 protein-coding genes (*ND1*, *ND2*, *COI*, *COII*, *ATP8*, *ATP6*, *COIII*, *ND3*, *ND4L*, *ND4*, *ND5*, *ND6*, and *Cyt b*), 22 transfer RNA (tRNA) genes, 2 ribosomal RNA genes, and a control region (Figure 1). The outermost layer of Figure1 is gene structure, where orange-yellow indicates the rRNA genes, orange-red indicates the tRNA genes, light green indicates the 13 protein-coding genes, and the remainder is the control region. Most genes are transcribed from the heavy strand (2 rRNAs, 12 protein-coding genes and 15 tRNAs); only eight genes, including one protein-coding gene (*ND6*) and seven tRNAs (*trnQ*, *trnA*, *trnN*, *trnC*, *trnY*, *trnS* and *trnE*), are encoded on the light strand. Notably, the *trnP* gene is encoded on the heavy strand instead of its typical light strand position, providing an example of gene inversion in vertebrate mitogenomes. *D. micangshanensis* shares the same gene arrangement type (inverted *trnP* gene) with other Draconinae species, indicating a single occurrence of the *trnP* inversion in the ancestral draconine lineage (Liu et al. 2019).

As is the case with other agamid mitogenomes, the overall base composition of *D. micangshanensis* is 34.1% A, 23.64% T, 13.62% C, and 28.64% G, with a slight AT bias of 57.74%. There are 11 overlapping regions totaling 40 bp (varying from 1 to 10 bp) and 14 intergenic spacer regions totaling 100 bp (varying from 1 to 33 bp). Almost all protein-coding genes (PCGs) start with the typical ATA/ATG initiation codons whereas *ATP8* starts with GTG. Most PCGs are terminated with the typical TAA/TAG/AGG/AGA codons, except for *ATP6*, *COIII*, *ND3*, and *Cyt b*, which are characterized by incomplete stop codons (T or TA). The 22 tRNA genes are interspersed along the genome, with the length varying from 51 to 75 bp. The 12S and 16S rRNA genes are 847 and 1496 bp, respectively. They are located between *trnF* and *trnL* (uaa) and are separated by *trnV* (Table 1). The D-loop region is located between *trnP* and *trnF*.

**Table 1.** Characteristics of 37 genes in the mitochondrial genome of *Diploderma micangshanensis*.

				Codon			
Gene/Element	From	To	Length (bp)	Start	Stop	Intergenic nucleotides*	Strand^†^
*trnF*	258	324	67			–1	H
s-rRNA/ 12S rRNA	324	1170	847			–2	H
*trnV*	1169	1234	66			–1	H
I-rRNA/16S rRNA	1234	2729	1496			+33	H
*trnL* (uaa)	2763	2837	75			+3	H
*ND1*	2841	3809	969	ATG	TAG	–5	H
*trnQ*	3805	3876	72			+2	L
*trnI*	3879	3949	71			0	H
*trnM*	3950	4014	65			0	H
*ND2*	4015	5043	1029	ATA	TAG	–2	H
*trnW*	5042	5113	72			+3	H
*trnA*	5117	5184	68			+9	L
*trnN*	5194	5265	72			+21	L
*trnC*	5287	5337	51			0	L
*trnY*	5338	5400	63			0	L
*COI*	5401	6978	1578	ATG	AGA	−5	H
*trnS* (uga)	6974	7044	71			+2	L
*trnD*	7047	7115	69			+3	H
*COII*	7119	7805	687	ATG	AGG	+6	H
*trnK*	7799	7865	67			+1	H
*ATP8*	7867	8028	162	GTG	TAA	–10	H
*ATP6*	8019	8701	683	ATG	T––	–1	H
*COIII*	8701	9485	785	ATG	TA–	–1	H
*trnG*	9485	9551	67			+4	H
*ND3*	9556	9892	337	ATG	T––	0	H
*trnR*	9893	9963	71			0	H
*ND4L*	9964	10,254	291	ATG	TAA	–7	H
*ND4*	10,248	11,615	1368	ATG	AGG	+7	H
*trnH*	11,623	11,685	63			0	H
*trnS* (gcu)	11,686	11,743	58			+4	H
*trnL* (uag)	11,748	11,818	71			0	H
*ND5*	11,819	13,597	1779	ATA	TAA	–4	H
*ND6*	13,594	14,100	507	ATG	TAG	0	L
*trnE*	14,101	14,168	68			+2	L
*cyt b*	14,171	15,303	1133	ATG	T––	–1	H
*trnT*	15,303	15,370	68			0	H
*trnP*	15,371	15,437	67			0	H

**Figure 2.**
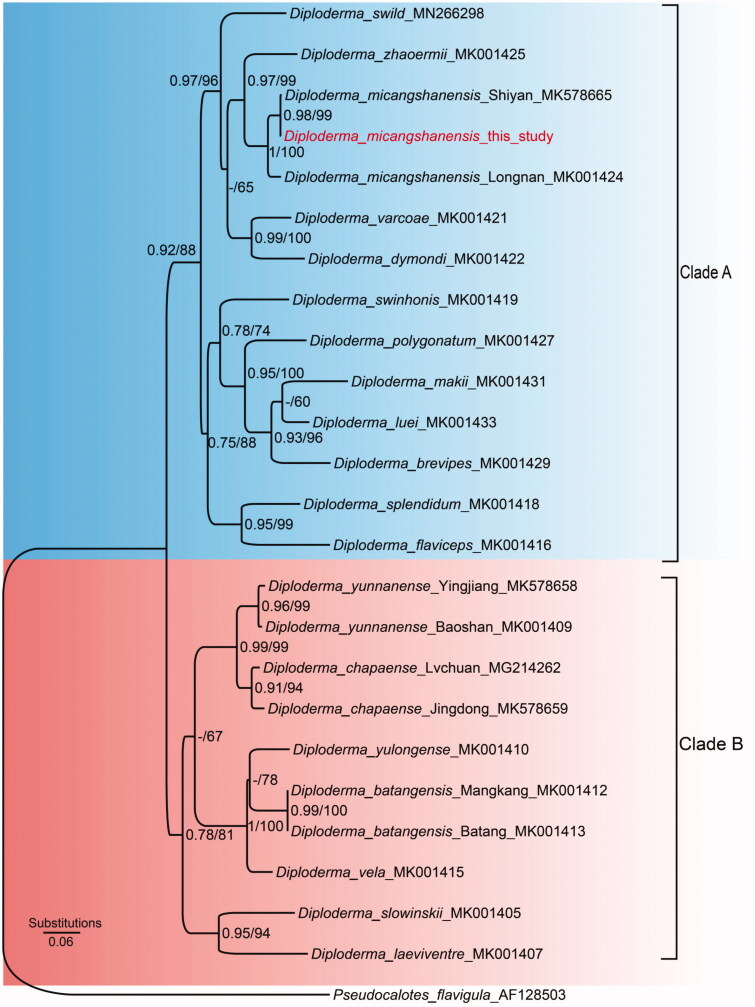
Phylogenetic relationships of species in genus *Diploderma* inferred by Bayesian Inference and Maximum Likelihood analyses, based on the mitochondrial *ND2* gene fragment. Numbers on the branches from left to right are Bayesian posterior probabilities obtained by BI and ML bootstrap values, respectively. Posterior probabilities less than 0.60 and bootstrap values under 60% are not shown.

Genetic distance shows the *D. micangshanensis* in this study has the closest distance with the *D. micangshanensis* deposited on NCBI (Table S1), which confirms that the sequenced specimen in this study indeed belongs to *D. micangshanense*. The two methods (BI and ML) generated a consistent phylogenetic topology that *D. micangshanensis* in this study clustered together with the individuals deposited in GenBank and displayed a closest relationship with *D. zhaoermii* (Figure 2). Additionally, the topology of *Diploderma* divide into two major clades (Clade A and B in Figure 2), and the *D. micangshanensis* locate into Clade A. The placement of *D. micangshanensis* was also supported by Wang et al. 2019. Thus, our study further verify and cofirm the phylogenetic position of *D. micangshanensis* with molecular data. In summary, our study provides a new resource for understanding whole mitochondrial genome of *D. micangshanensis*, which will promote the molecular study on this species.

## Data Availability

The genome sequence data that support the findings of this study are openly available in GenBank of NCBI at (https://www.ncbi.nlm.nih.gov/) under the accession no. MW242820. The associated BioProject, SRA, and Bio-Sample numbers are PRJNA675949, SRR13022469, and SAMN16736307 respectively.
